# Effect of dietary patterns on dental caries among 12–15 years-old adolescents: a cross-sectional survey

**DOI:** 10.1186/s12903-023-03566-y

**Published:** 2023-11-09

**Authors:** Xiangyu Wang, Hao Chen, Ruxia Hou, Tingting Yang, Jiajia Liu, Junming Li, Xiaotong Shi, Bin Zhao, Junyu Liu

**Affiliations:** 1 Department of Pediatric and Preventive Dentistry, School and Hospital of Stomatology, Shanxi Medical University, Taiyuan, 030001 China; 2 Shanxi Province Key Laboratory of Oral Diseases Prevention and New Materials, Taiyuan, 030001 China; 3 School of Statistics, Shanxi University of Finance and Economics, 696 Wucheng Road, Taiyuan, 030006 China; 4 School and Hospital of Stomatology, Shanxi Medical University, Taiyuan, 030001 China

**Keywords:** Dental caries, Risk factors, Dietary pattern, Social determinants of health, Adolescents, Cluster analysis

## Abstract

**Background:**

Several factors can influence the risk of dental caries, among which dietary factors have a significance impact on the occurrence of dental caries. The limitation of current studies is that they only focus on the influence of individual foods on the risk of dental caries. This study use cluster analysis to examine the relationship between dietary patterns and dental caries experience among adolescents aged 12–15.

**Methods:**

Based on data from the first oral epidemic survey in Shanxi Province, a cross-sectional study was conducted among 11,351 adolescents aged 12–15 in Shanxi Province through oral examination and questionnaires. The questionnaire included the intake frequency of seven types of food. Descriptive statistics, cluster analysis, and multinomial logistic regression were used to analyze the association between dietary patterns and dental caries experience.

**Results:**

The prevalence rate of caries was 44.57% and the mean DMFT score was 0.98 ± 1.49 in adolescents aged 12–15 in Shanxi Province. The caries rate was higher in females than males (*X*^2^ = 103.59, *P* < 0.001). Adolescents who grow up in one-child families have a lower caries risk than those who grow up in families with more than one child (OR:0.91; 95%CI:0.84–0.97). The dietary patterns of adolescents aged 12–15 can be divided into eight types, among which refreshments-rich diet (OR:1.47; 95%CI,1.22–1.77) can increase the risk of caries, while the coarse-grains-rich dietery pattern (OR:0.90; 95%CI, 0.79–0.97) has a lower caries risk.

**Conclusions:**

Social determinants of health such as sex, family size and dietary patterns influence the risk of dental caries. Certain dietary patterns could increase or decrease the risk of caries. The government, school canteens and news media should take dietary pattern factors seriously.

**Supplementary Information:**

The online version contains supplementary material available at 10.1186/s12903-023-03566-y.

## Background

Dental caries is a prevalent and widely distributed tooth hard tissue diease. According to the World Health Organization, it is one of the most common diseases, along with cardiovascular disease and tumors, that must be prevented and controlled [1, 2]. In the US, 27% of individuals under the age of 64 have untreated tooth decay, while 91% of adults have dental caries [3]. The cost of preventing and treating caries is a global economic burden [2]. For instance, the projected spending for treating dental disease was $122 billion in 2014 (Centers for Medicare and Medicaid Services 2011) [3].

Caries risk is influenced by multiple factors, including biological factors [4–7] and abiotic factors (such as deciduous dental caries [8], family influences [9], socio-economic status [10], BMI [11], and dietary habit [12]). Scientific and epidemiological data suggest a lifelong synergy between diet, nutrition, and the integrity of the oral cavity in health and disease [13]. Higher diet quality is related to a lower index of decayed, missing, and filled teeth (DMFT) [14]. It is currently accepted that caries is a sugar and biofilm-dependent disease. The pathogenesis is well understood: bacteria in dental plaque (biofilm) metabolize dietary sugars to acids that dissolve dental enamel and dentine [15]. Plaque bacteria produce acids from the metabolism of fermentable carbohydrates that lead to the demineralization of tooth enamel and enzymes that attack the protein component of the tooth, resulting in decay. Sugar is commonly found in bread, chocolate, drinks, fruits, etc. An unhealthy diet will cause people to consume excessive sugar. Moreover, other dietary factors besides sugar can also affect caries. For example, one study showed that a lack of vitamin D in the diet increased the risk of dental caries [16]. The intake of milk can reduce the caries risk to a certain extent [17]. Therefore, it is of great significance to reveal the association between diet and caries for reducing caries risk.

Due to the significant influence of diet on caries risk, researchers worldwide have conducted quite a few studies in recent years. However, the frequency of eating may be more closely related to the experience of caries than the amount of food consumed [18, 19]. A study of 4,467 individuals in the United States, using a principal component analysis of data from a 24-h dietary recall, reduced eating patterns to three and found that a diet of "high-sugar drinks and sandwiches" was associated with the prevalence of DMFT [20]. Another analysis of dietary frequency studies found that American children who ate fewer than five servings of fruits and vegetables a day were more likely to develop cavities in their primary teeth (OR = 3.21, *P* < 0.05) [21]. Different foods have bidirectional effects on the occurrence of caries. A study of 4,111 New Zealand children found that higher consumption of ice cream, noodles, rice porridge and refined breakfast cereals was positively associated with dental caries [17]. At the same time, some studies have revealed a positive effect of specific diets on caries risk. A novel analysis from Sanders and colleagues represented that an increased intake of long-chain omega-3 fats, whole grains, and vegetables (excluding potatoes) was inversely associated with dental caries [22]. Related research has also been carried out in China. Qin conducted a study on caries in children aged 10–12 in Guangdong province, showing that those who ate desserts or chocolate more than twice a day were more likely to have caries [23]. Those who ate sweets at least once a day had 69.8% of dental caries, compared with 57.5% for those who ate sweets less than once a day (*P* = 0.027) [24].

The researchers used many statistical analysis methods to explore the potential association between dietary patterns and caries experience. Multinomial logistic regression was used to study the influencing factors of dental caries among adolescents in Guangdong province by Li and colleagues, and found that a high frequency of sweet milk, tea, or coffee was a risk factor for dental caries [24]. In the meantime, a binary logistic regression model showed that the caries prevalence was not associated with cake or dessert intake but with flavored milk or yogurt and honey [25]. Although these studies effectively demonstrate a potential link between the frequency of consumption of certain foods and the caries prevalence, a person's eating habits are composed of many food types [26]. There are complex interactions and potentially cumulative relationships between foods [27]. Moeller argues that it is not advisable to study the separate effects of each diet on disease [28]. Schulze 's study also showed that if studying the effect of a single food on disease separately, the effect of a single food on disease separately is difficult due to multicollinearity [29]. Compared with traditional statistical analysis methods, the cluster analysis method has an advantage in solving this problem. It can stratify samples into diverse groups according to individual characteristics [30, 31].

The up-to-date study in China lacked a comprehensive analysis of the relationship between dietary patterns and caries experience. The study on the relationship between dietary patterns and dental caries experience is beneficial in guiding adolescents to improve their dietary structure and reduce the risk of dental caries. As a result, this study used descriptive statistics and cluster analysis to analyze the caries experience of adolescents aged 12–15 in different areas of Shanxi Province. The aim of this study is to reveal the potential link between the dietary patterns of adolescents and the risk of dental caries, thus guiding for the government and school canteens to improve policies and citizens' nutritional habits in the future.

## Methods

### Study design and participants

This survey was conducted with reference to the WHO Oral Health Surveys [32], the Basic Methods (5th ed), and the Oral Health Survey Test Methods issued by the former National Health and Family Planning Commission in 2015. The data of this study were based on the first oral epidemic survey in Shanxi Province in 2018. The protocol was reviewed and approved by the Ethics Committee of Shanxi Medical University. All the guardians of the investigated personnel signed informed consent.

**Fig.1:**
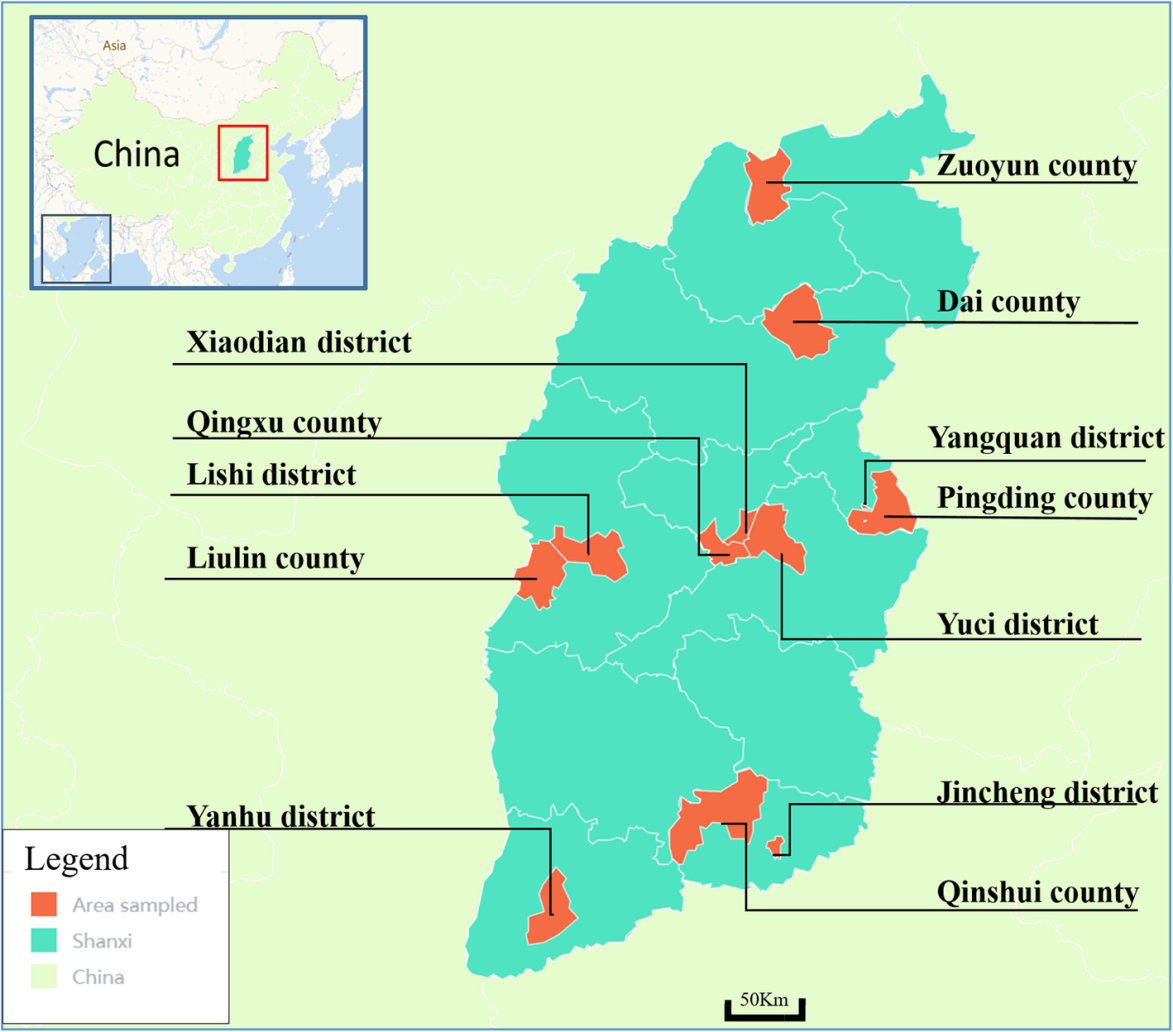
The randomly sampled areas. (Compiled from the base map provided by Amap: https://lbs.amap.com/)

According to the characteristics of regional distribution and the principle of random sampling, 117 municipal districts, counties, and county-level cities in 11 administrative districts of Shanxi Province were sampled. The method of stratified random sampling was used to select samples from participants. We collected the list of middle schools in the survey districts (county/county-level city) and ranked middle schools according to the size of students aged 12–15. Probability Proportionate to Size Sampling (PPS) was used to select three middle schools in each city (county/district). We have selected a total of 36 schools in 12 cities (counties/districts) through multi-level stratified random sampling, taking into account the balance of regional and urban–rural distribution as well as the feasibility of implementation (Fig. 1). The sample size is calculated bases on the formula:

$$n=\frac{{Z}_{1-\alpha /{2}^{\times }}^{2}p(1-p)}{{\delta }^{2}}$$

where P is the dental caries rate among 12-year-olds according to the Fourth National Oral Health Epidemiological Survey in China [35], P = 34.5%. α = 0.05, δ = 0.1, and the Z value can be obtained by checking the normal distribution table: Z = 1.96. The unit nonresponse rate is calculated at 10%. The sampling design efficiency is deff = 4. The result is 95 students per age per school (*n* = 95),Therefore *N* = n*36*4 = 13,680. Ultimately, the sample included 13,680 individuals from 36 schools in 12 different cities (counties/districts) in Shanxi Province, China, who aged 12 to 15 and had lived locally for at least six months.

### Data collection and data quality assurance

All dentists received theoretical knowledge training and passed the standard compliance test in early 2018 (kappa value greater than 0.8). The lead team for the epidemiologic investigation provided certificates to the examiners before their participation in the epidemiological investigation. The examiners performed oral examinations using WHO standards and disposable dental mirrors. They recorded the DMFT of each tooth in detail and conducted reexamination for a random 5% of samples on a daily basis. Caries diagnostic criteria for decayed, missing, and filled teeth (DMFT) were used to estimate dental caries prevalence.

### Questionnaire

Each participant was asked to independently complete a questionnaire before undergoing an oral health examination. The questionnaire included basic information such as gender, date of birth, region, ethnic, and so on. Importantly, the questionnaire covered the frequency of consumption of the following foods:

- Desserts and confectionery (biscuits, cakes, bread, chocolate, sugary mouth candy);
- Sweet drinks (sugar water, carbonated drinks such as cola, fruit juices such as orange juice and apple juice, non-fresh fruit juices such as lemonade);
- Sugar-sweetened milk (yogurt, milk powder, tea, soy milk, coffee);
- Vegetables;
- Fruits;
- Coarse grains (corn, purple rice, sorghum, oats, buckwheat, wheat bran);
- Protein foods (beans, eggs, meat, fish, animal offal).

Options for each meal questionnaire were rated on a scale of six, ranging from "Never or hardly ever" to "more than once a day". The questionnaire that answered at least 80% of all questions and 100% of the key questions is considered to be the complete interview. After the preliminary design of the questionnaire, we carried out a pretest. The results of reliability analysis showed that the Cronbach's coefficient α is 0.82 which means the questionnaire has good reliability.

### Additional covariates

The covariates associated with caries affected the outcome. Therefore, they need to be analyzed in statistical analysis to try to remove their effect. Our study identified potential confounders based on prior literature. Thus, in addition to the above questions, the questionnaire also included the following items: family size (one-child or more-than-one-child family), residence place (rural and urban), ethnicity, and brushing habits (ranging from "never or hardly ever" to "more than twice a day"). By querying the local official website, we collected the following information: Normalized Difference Vegetation Index (NDVI) [33], mean annual precipitation, mean annual temperature, PM2.5 (Statistical Yearbook of Shanxi Province,2017), and Digital Elevation Model (DEM, Data based on ASTER GDEM).

### Statistical analysis

This study used simple descriptive statistics (mean and ratio) for data processing. The chi-square test, t-test, and analysis of variance (ANOVA) were used to analyze the validity of the results. Multinomial logistic regression and cluster analyses were used to study diet and caries risks. P values of 0.05 or less were considered statistically significant. The researchers used multiple Microsoft Access entries to enter all data. SPSS 26.0 statistical software was used to analyze the data.

Based on monthly intake frequency, the scores of 7 dietary questionnaires were assigned proportionally to evaluate the relative effect of dietary intake frequency on the existence of dental caries. (Table 1) This assignment cannot accurately represent the actual frequency of food intake each month. However, we believe that this way of assigning values can still reflect the relative ratio of intake foods frequency.

**Table 1:** Frequencies assignment table

Item	Assignment
Never or hardly ever	0
1–3 times a month	2
once a week	4
2–6 times a week	16
Once a day	30
More than once a day	60

K-means clustering analysis was used to explore the dietary characteristics of valid samples. The statistical process of the k-means clustering algorithm is to divide N samples into K clusters so that the sample points within each group have high similarity. In contrast, the sample points between groups have low similarity. The similarity degree is calculated according to a cluster's average value of sample points. The theorem is to use the Euclidean distance as the similarity index, repeatedly calculate the clustering center, divide the data with high similarity into the same category, and finally divide all the samples into K clusters. Therefore, the initial K value is a crucial parameter, and determining the optimal K significantly influences the statistical results. In this study, we used the "elbow" method to determine the optimal K value, which is implemented by calculating the sum of squares of error (SSE):

$${\text{SSE }} = \sum\limits_{{{\text{i}} = 1}}^{{\text{k}}} {\sum\limits_{{{\text{j}} \in {\text{C}}_{{\text{i}}} }}^{{}} {\left| {{\text{j - }}\overline{C}_{{\text{i}}} } \right|^{2} } }$$

In this formula, SSE represents the error value, and the C_i_ is any one cluster. The j represents the samples contained in this cluster. *C*_i_ symbolizes the center of this cluster. For a cluster, the lower the SSE, the closer cluster members are to each other. In fundamental respects, SSE decreases as the number of clusters (K value) increases. But when a critical point is reached, the distortion drops sharply and afterward slowly. This tipping point, known as the "elbow", can be considered the point of the best clustering performance. The maximum number of iterations is set to 50. The convergence criterion is 0, and The initial cluster centers are not disturbed in the k-means clustering analysis.

## Results

### Participant characteristics

A total of 13,680 adolescents aged 12 to 15 years old in Shanxi Province were surveyed, of which the proportion of complete interview was 88.02%, the proportion of partial interview was 8.11% and that of break-off interview was 3.87%. After removing those who were missing or unrecorded in the clinical examination, the final valid sample consisted of 11,351 individuals. Among them, 11,329 were Han Chinese, accounting for 99.8%. The response rate in this cross-sectional study was 82.98%.

### Descriptive statistics

The caries prevalence and associated factors are shown in Table 2. The caries prevalence rate among adolescents aged 12 to 15 in Shanxi is 44.57%. The caries prevalence in males (39.81%) was lower than in females (49.31%). Females aged 12 to 15 in Shanxi, China, are more susceptible to dental caries than males, with statistically significant differences (*X*^2^ = 103.59, *P* < 0.001). The DMFT score in Shanxi Province was 0.98 ± 1.49. In adolescents aged 12 ~ 15 years, the caries rate was positively correlated with age, and the difference was statistically significant (*X*^2^ = 25.33, *P* < 0.01).

**Table 2:** Dental caries prevalence and associated factors (*N* = 11,351)

Characteristics	Total (n/N^a^100)	Caries prevalence (%)		*X*^*2*^*/P*^1^	DMFT^2^ (x ± s)	*P*^3^
		Without Caries	With Caries			
**Sex**						
Male	5662(49.88)	3408(60.19)	2254(39.81)	103.59/ < 0.001	0.81 ± 1.30	< 0.001
Female	5689(50.12)	2884(50.69)	2805(49.31)		1.14 ± 1.65	
**Age**						
12	2848(25.09)	1680(58.99)	1168(41.01)	25.33/ < 0.05	0.82 ± 1.29	< 0.01
13	2799(24.66)	1570(56.09)	1229(43.91)		0.89 ± 1.33	
14	2858(25.18)	1529(53.50)	1329(46.50)		1.05 ± 1.56	
15	2846(25.07)	1513(53.16)	1333(46.84)		1.14 ± 1.71	
**Region**						
North Shanxi	1848(16.28)	900(48.70)	948(51.30)	43.26/ < 0.001	1.23 ± 1.67	< 0.001
Middle Shanxi	6627(58.38)	3723(56.18)	2904(43.82)		0.97 ± 1.49	
South Shanxi	2876(25.34)	1669(58.03)	1207(41.97)		0.84 ± 1.33	
**Residence place**						
Urban	5668(49.93)	3180(56.10)	2488(43.90)	2.08/ 0.15	0.94 ± 1.46	0.009
Rural	5683(50.06)	3112(54.76)	2571(45.24)		1.01 ± 1.53	
**Brushing teeth habit**						
Never or hardly ever	1016(8.95)	418(41.14)	598(58.86)	101.79/ < 0.001	1.56 ± 2.02	< 0.001
Not every day	1090(9.60)	577(52.94)	513(47.06)		1.23 ± 1.88	
Once or twice a day	3167(27.90)	1782(56.27)	1385(43.73)		1.01 ± 1.57	
More than twice a day	6078(53.55)	3515(57.83)	2563(42.17)		0.82 ± 1.21	
**Family size**						
One child	3291(28.99)	1897(57.64)	1394(42.36)	9.17/ 0.02	0.933 ± 1.48	0.044
More than one child	8060(71.01)	4395(54.53)	3665(45.47)		0.995 ± 1.50	
**Total**	11,351(100)	6292(55.43)	5059(44.57)		0.98 ± 1.49	

^1^Chi-square test

^2^After testing, the data of each group conformed to the normal distribution (*P* > 0.05)

^3^t-test and ANOVA

**Fig. 2:**
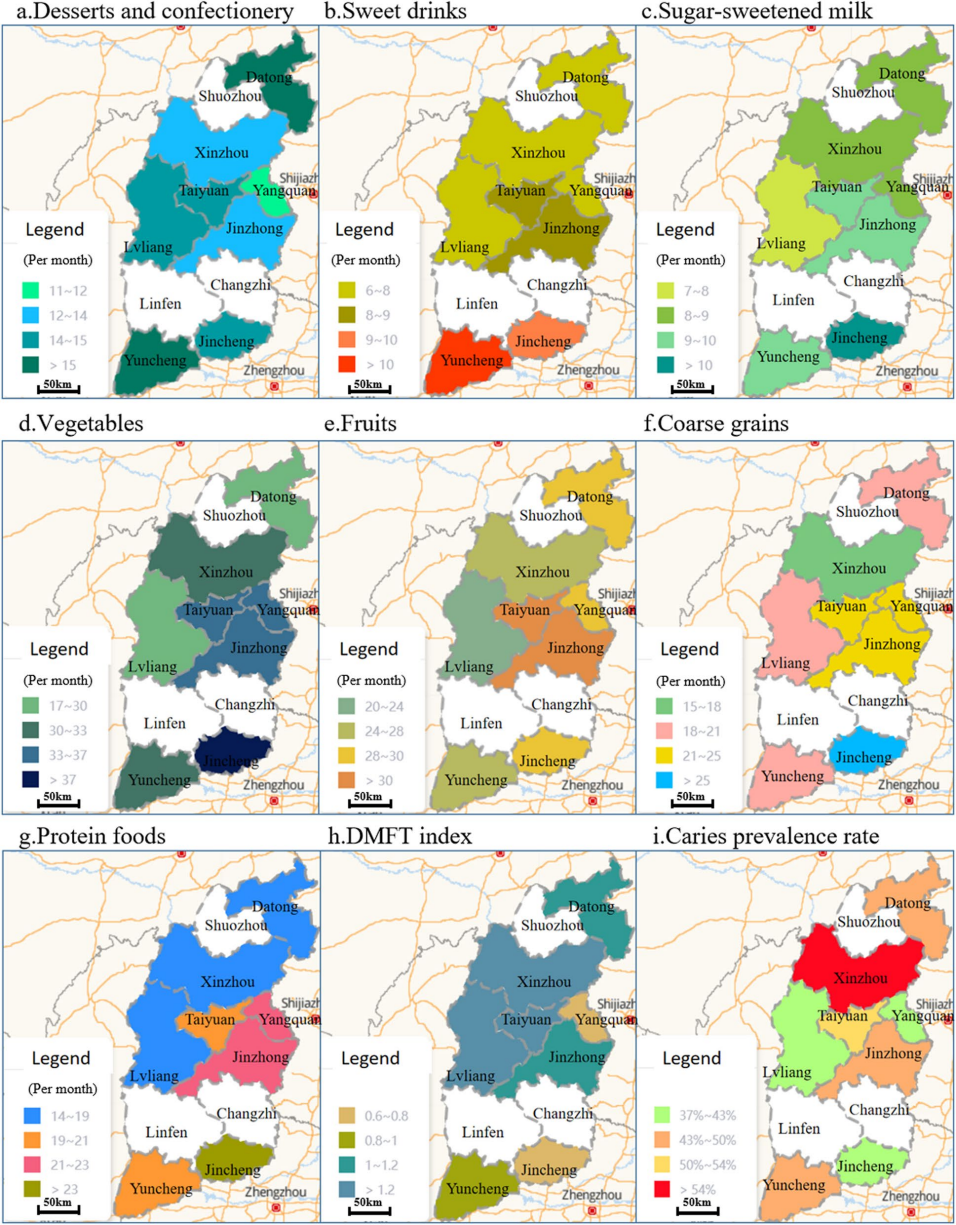
Geographical distribution* of dietary questionnaires (monthly intake frequency) and caries prevalence. (Compiled from the base map provided by Amap: https://lbs.amap.com/)*To clearly show the comparison situation, the schematic scope of the surveyed areas in the figure is expanded to the administrative cities (counties) where it is located

As shown in Fig. 2, There are differences in eating habits among different regions in Shanxi. For example, the dessert consumption frequency in Zuoyun county, Datong, and Yanhu District, Yuncheng, is relatively higher than in other regions. The average prevalence of caries decreased from North to South in Shanxi Province, with the highest prevalence in Xinzhou (1.39) and the lowest prevalence in Yangquan (0.75), showing a significant difference (P < 0.05).

### Dietary patterns and caries analysis

**Fig. 3:**
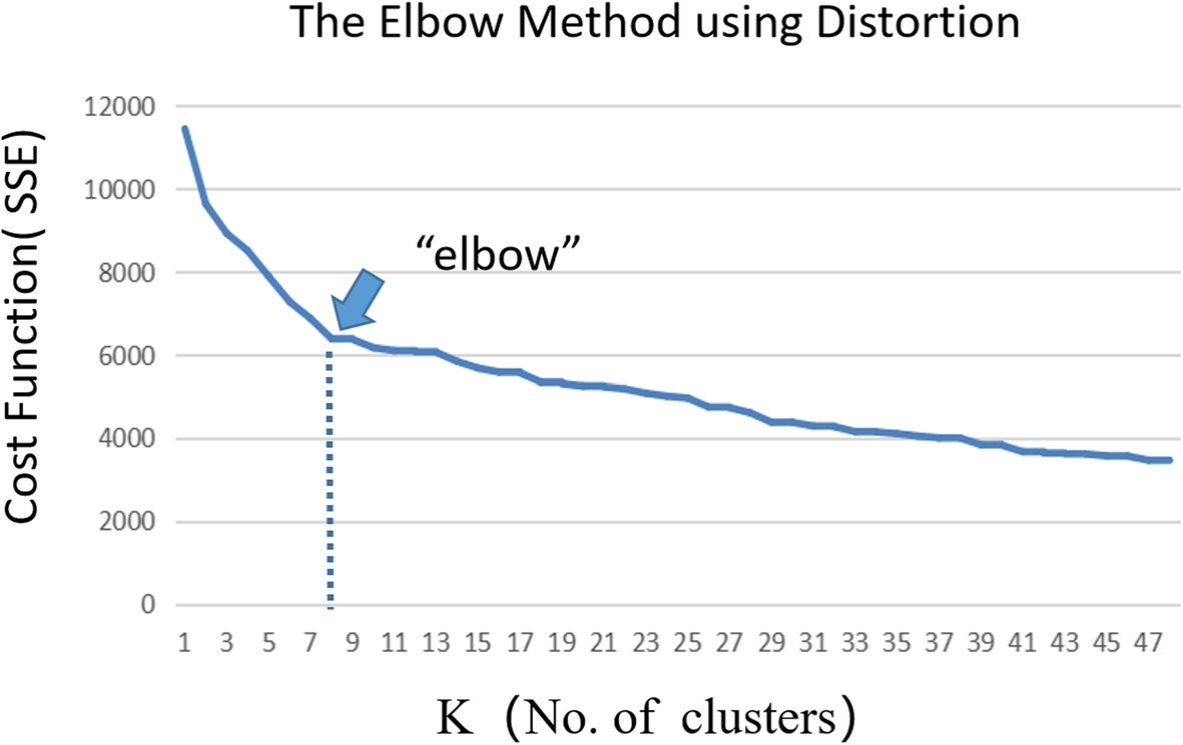
Trend chart of SSE value under different K values

Our study used the "elbow" method for determining the number of classifications (Fig. 3). The analysis result is that when the k value is 8 (that is, when the sample is divided into eight categories), it is considered to be the optimal classification number statistically. The cost function is relatively small at this point, and the k value is conducive to the research.

According to the characteristics of individual diet frequency, eight dietary cluster groupings were obtained by the k-means clustering algorithm. Secondarily according to the features of the final cluster center, we named the cluster groupings as eight different diet patterns (Table 3). There were statistical differences among all groups (*P* < 0.001). For the convenience of description, we name them the following eight categories:

- 1:High balanced frequency diet. (The eating frequency of all kinds of special food is relatively high.)
- 2:Low balanced frequency diet. (The eating frequency of all kinds of special food is relatively low.)
- 3:Non-staple-food-rich diet. (The eating frequency of fruits and vegetables is relatively high)
- 4:Refreshments-rich diet. (The eating frequency of all kinds of sweet foods is relatively high)
- 5:Vegetable-rich diet
- 6:Coarse-grains-rich diet.
- 7:Limited-refreshments diet. (The eating frequency of other foods except sweets is relatively high)
- 8:Desserts-rich diet.

**Table 3:** Distribution of food intake frequency in the dietary patterns (Dietary K-means clustering analysis, final cluster center)

Item	Total (*N* = 11,351)	High balance frequency diet (*n* = 495)	Low balance frequency diet (*n* = 495)	Non-staple-food-rich diet (*n* = 1,25)	Refreshments-rich diet (*n* = 521)	Vegetable-rich diet (*n* = 1,339)	Coarse-grains-rich diet (*n* = 1,752)	Limited refreshments diet (*n* = 1,125)	Desserts-rich diet (*n* = 753)
Desserts and confectionery	14.04	53.56	6.91	8.82	37.01	9.55	8.53	9.33	47.61
Sweet drinks	8.19	31.05	4.68	5.59	38.96	5.87	4.95	5.67	10.79
Sugar-sweetened milk	8.95	30.07	5.26	7.55	34.05	7.5	7.04	8.49	7.85
Vegetables	33.96	54.8	16.68	48.91	20.03	59.42	30.76	56.41	27.93
Fruits	27.22	50.71	13.85	60	24.2	19.58	21.34	51.49	23.2
Coarse grains	22.43	47.37	7.96	19.07	17.41	14.15	41.65	55.54	14.64
Protein foods	20.05	38.97	9.93	17.93	23.06	20.56	22.62	47.96	15.64

More adolescents were grouped into the “low balanced frequency diet” cluster (*n* = 4,110), while “high balanced frequency diet” cluster had the fewest individuals (*n* = 495).

The prevalence of dental caries was the highest among adolescents with the refreshments-rich diet (52.78%), while that of coarse-grains-rich diets was the lowest (42.01%). Adolescents with a higher DMFT score were prone to have dietary patterns of high balance frequency, refreshments-rich diet and desserts-rich diet, while those in coarse-grains-rich diet group had lower DMFT score. In South Shanxi, the caries prevalence among adolescents with the vegetable-rich diet was the lowest, which was merely 37.89%, lower than that in North Shanxi (*P* < 0.05). The proportion of individuals with limited-refreshments dietary habits in North Shanxi was less than that in other regions, and the DMFT score was higher than that in Middle and South Shanxi (*P* < 0.05) (Table 4).

**Table 4:** ANOVA of dietary patterns in different areas

Regions	*N*	High balanced frequency diet(A)	Low balanced frequency diet(B)	Non-staple-food-rich diet(C)	Refreshments-rich diet(D)	Vegetable-rich diet(E)	Coarse-grains-rich diet(F)	Limited refreshments diet(G)	Desserts-rich diet(H)
North Shanxi (a)									
n(n/N*100%)	1848(100)	63(3.41)	771(41.72)	252(13.64)	93(5.03)	152(8.23)	241(13.04)	100(5.41)	176(9.52)
Number of caries (Caries rate,%)	948(51.3)	37(58.73)	373(48.38)^Hbc^	122(48.41)^H^	52(55.91)	82(53.95)^bc^	124(51.45)^Hbc^	47(47.00)^H^	111(63.07)^BCFGbc^
DMFT index	1.23	1.6^Bc^	1.11^ADEHbc^	1.15^bc^	1.51^Bc^	1.47^Bbc^	1.14^bc^	1.34^bc^	1.39^Bc^
Middle Shanxi (b)									
n(n/N*100%)	6627(100)	276(4.16)	2395(36.14)	715(10.79)	277(4.18)	770(11.62)	1094(16.51)	694(10.47)	406(6.13)
Number of caries (Caries rate,%)	2904(43.82)	129(46.74)	1032(43.09)^Da^	321(44.90)^D^	148(53.43)^BCEFG^	327(42.47)^Da^	442(40.40)^DGa^	315(45.39)^DF^	190(46.80)^Fa^
DMFT index	0.97	1.24^BCEFG^	0.96^ADFHac^	0.94^ADHa^	1.17^BCEFG^	0.93^ADHa^	0.84^ABDHa^	0.93^ADHa^	1.17^BCEFG^
South Shanxi (c)									
n(n/N*100%)	2876(100)	156(5.42)	944(32.82)	289()10.05	151(5.25)	417(14.50)	417(14.50)	331(11.51)	171(5.95)
Number of caries (Caries rate,%)	1207(41.97)	79(50.64)^BEF^	383(40.57)^ADa^	124(42.91)	75(49.67)^BE^	158(37.89)^ADa^	170(40.77)^Aa^	139(41.99)	79(46.2)
DMFT index	0.84	0.99	0.83^ab^	0.88^a^	1.04^FGa^	0.81^a^	0.77^Da^	0.76^Da^	0.95^a^
Total									
n(n/N*100%)	11351(100)	495(4.39)	4110(36.21)	1256(11.07)	521(4.59)	1339(11.80)	1752(15.43)	1125(9.91)	753(6.63)
Number of caries (Caries rate,%)	5059(44.57)	245(49.49)^BEF^	1788(43.50)^ADH^	567(45.14)^DH^	275(52.78)^BCEFG^	567(42.35)^ADH^	736(42.01)^ADH^	501(44.53)^DH^	380(50.46)^BCEFGa^
DMFT index	0.98	1.21^BCEFGa^	0.96^ADFH^	0.97^ADH^	1.19^BCEFG^	0.96^ADH^	0.86^ABDH^	0.92^ADH^	1.17^BCEFG^

^A-H^Assuming the homogeneity of variance, the significant difference in the value of the corresponding letter column marks in the same line (*P* < 0.05)

^a-c^Assuming the homogeneity of variance, the significant difference in the corresponding regional target value in the same column (*P* < 0.05)

Table 5 shows the results of multiple logistic regression after adjusting for covariates. The results indicate that compared to the low balanced frequency diet, both the high balanced frequency and refreshments-rich dietary patterns could increase the caries risk (*P* < 0.05). The caries risk in males was 0.691 times that in females (OR:0.69; 95%CI,0.64–0.75).

**Table 5:** Association between caries prevalence and dietary patterns after adjusting for covariates^1^

Variable	OR	95% CI (Lower–Upper)
Dietary patterns		
High balanced frequency diet	1.23^a^	1.02–1.49
Non-staple-food-rich diet	1.03	0.91–1.18
Refreshments-rich diet	1.47^b^	1.22–1.77
Vegetable-rich diet	0.95	0.86–1.06
Coarse-grains-rich diet	0.90^a^	0.79–0.97
Limited refreshments diet	1.07	0.92–1.25
Desserts-rich diet	1.28^b^	1.09–1.50
Low balanced frequency diet	——	——
Sex		
Male	0.69^b^	0.64–0.75
Female	——	——
Age		
12	0.74^b^	0.66–0.82
13	0.85^a^	0.77–0.95
14	0.97	0.87–1.08
15	——	——
Residence place		
Urban	0.87	0.46–1.66
Rural	——	——
Brushing teeth habit		
Never or hardly ever	1.95^b^	1.69–2.24
Not every day	1.23^b^	1.10–1.39
Once a day	1.08	0.98–1.17
At least twice a day	——	——
Family		
one child	0.91^a^	0.84–0.97
More than one child	——	——

^1^*OR* Odds ratio, *CI* Confidence interval, Other adjusting confounders: NDVI, mean annual temperature, mean annual precipitation, DEM, PM2.5

^a^It is statistically significant on a 95% confidence interval

^b^It is statistically significant at a 99% confidence interval

## Discussion

Adolescent aged 12 to 15 years are an intensely vital group for being studied in epidemiological surveys of caries. At this age, all their permanent teeth (excluding the third molars) have erupted, and they begin to independent determine their own diet and oral hygiene [34]. Therefore, for this cross-sectional survey conducted in 2018, adolecsents aged 12 to 15 were selected as the subjects for both a questionnaire and oral health examination.

The prevalence of dental caries in mainland China has generally high from 1980s (52.0%,95% CI:49.4%-54.6%)to 2010s (53.1%,95% CI:50.8%-55.5%), showing an increasing trend over the past 38 years [35]. The fourth oral epidemiological survey in China showed that the caries rate of permanent teeth among adolescents aged 12–15 years was 41.9%, and the DMFT score was 1.04 [36]. In our study, the caries rate of permanent teeth in Shanxi adolescents aged 12–15 years was significantly higher than the national average (*P* < 0.05) and lower than that of Hainan Province (60.5%) [37] and Liaoning Province (53.2%) [38]. The caries prevalence in 12–15-year-old males in Shanxi was relatively lower than that in females (*P* < 0.001), which was consistent with the Xiao [39] in Jiangsu Province. The result may be because female eat snacks more frequently than male. Additionally, our study revealed significant spatial heterogeneity in the prevalence of dental caries in different areas of Shanxi, consistent with latest study [40].

Caries was affected by many risk factors, among which dietary factors should not be ignored [41]. Our study found significant differences in the intake frequency habits of different foods in different regions. In this study, we divided the dietary types of adolescents aged 12 to 15 into eight categories through cluster analysis. According to their frequency distribution characteristics, we named eight different dietary patterns. The refreshment-rich diet has a higher OR value than the desserts-rich diet, which may be because the dietary patterns with a significantly higher frequency of sugary foods may have contributed to the increased caries risk. This conclusion is consistent with earlier studies [15, 17, 19]. A higher consumption of high-fibre grain products was associated with fewer caries [42]. Our study found that a diet rich in coarse grains seemed to slightly reduce the caries risk (OR:0.90;95%CI,0.79–0.97), which is consistent with the findings of Dye [21] and Thornley [17]. After adjusting for confounding factors, the risk of caries in the "vegetable-rich diet" group (OR:0.95;95%CI,0.86–1.06) was insignificant at a 95% confidence interval. This result may be due to the relatively small sample size of the study or the fact that the frequency of vegetable intake had a weak effect on caries risk [20]. Non-staple-food-rich dietary pattern has no significant difference compared with the low-balanced frequency diet. However, Guo's research shows that individuals with high caries activity have a low intake of fruits, vegetables, and fiber [43]. Adolescents with the high-frequency balanced diet had a higher risk of caries than those with a low-balanced frequency diet. This may be because the ability of sugary foods to increase the risk of caries was much greater than that of coarse grains and vegetables to reduce the risk of caries.

Oral health is influenced by social determinants of health (SDH), which predispose individuals and communities to greater risk of developing caries [44]. In addition to the age and sex, factors such as agriculture/food production, living environment, and family conditions also contribute to the SDH of dental caries. Our study found that the proportion of "coarse-grains-rich diet" cluster in South Shanxi is similar to that in North Shanxi. (*P* < 0.05) However, the caries prevalence and DMFT score of "coarse-grains-rich diet" cluster in South Shanxi were significantly lower than those in North Shanxi. The per capita meat output is higher in the south of Shanxi Province, resulting in a relatively high proportion of protein foods. Conversely, the proportion of "limited-refreshments diet" and "vegetable-rich diet" patterns in North Shanxi was relatively low, corresponding to the region's high caries rate and DMFT score. The climate in North Shanxi is fairly cold, with less precipitation. The planting area of sorghum, millet, corn, and other crops is rather large, while the corresponding output of vegetables and fruits was relatively small [45]. It may indicate that a higher frequency of refreshments, a low frequency of vegetable and fruit intake are essential reasons for the higher caries risk in North Shanxi. The caries prevalence in Middle Shanxi is slightly higher than in South Shanxi. Therefore, we speculate that the local per capita output of various foods might be one of the reasons for the difference of dietary patterns and caries experience. Additionally, adolescents who grow up in one-child families have a lower caries risk than those who grow up in families with more than one child. Kateeb [46] suggests that improving the socio-economic conditions would give parents more control over their children’s oral health and minimize the level of the disease, which also emphasizes the crucial influence of SDH.

Preventing adolescent oral diseases through improved dietary patterns, not only in Shanxi Province, but worldwide, requires the positive influence of individuals and social factors. The specific suggestion is that the government, school canteens and media outlets should advocate for a diet pattern with low caries risk. The government and society should encourage the reduction of the caries risk among adolescents, including but not limited to popularize the causes of caries occurrence and development, advocate for a healthy diet with low sugar content, implementing a tax on sugar-sweetened beverages (SSBs), and control the proportion of various nutritious foods in school canteens.

### Strength and limitations of the study

This cross-sectional survey represents the largest survey of oral diseases in Shanxi Province in the twenty-first century. Using cluster analysis, we investigated the association between dietary patterns and the risk of caries. This method is rarely used in studies examining dietary patterns and dental caries. It has the advantage of comparing individuals' dietary patterns as a whole rather than just focus on individual foods, and it produces clear and easily understandable results [25, 31, 47].

However, our study still has some limitations. First, although it is internationally recognized that eating frequency is more important than food intake for dental caries research, we only investigate dietary frequency and ignored the effect of nutritional intake on caries risk. Second, despite using the scientific sample estimation method to determine the sample size, after the dietary cluster analysis, certain clusters have a small number of individuals, which made it challenging to produce statistical significance in the confidence interval. Additionally, our study was a cross-sectional study. Cohort studies have an unparalleled advantage in exploring the link between diet and dental caries. For example, a prospective study found an association between early-life free sugar intake and the prevalence of dental caries five years later [48]. Therefore, we plan to conduct a cohort study to reveal further the potential link between dietary patterns and dental caries risk.

## Conclusions

This study reveals the relationship between dietary patterns and dental caries experience and analyzes several dietary patterns that can reduce or increase the risk of dental caries. Reducing the intake frequency of sugary food (desserts, confectionery and sugary drinks) and increasing the intake frequency of coarse grains can reduce the risk of dental caries in adolescents. Social determinants of health such as sex, family size, and dietary patterns influence the risk of caries. The government, school canteens and news media should take dietary pattern factors seriously.

### Supplementary Information

**Additional file 1.** Questionnaire for the first oral health epidemiological survey in Shanxi Province (Adolescents aged 12-15).

## Data Availability

Public access to the database is closed. However, the deidentified data set used and/or analyzed are available from the corresponding author on reasonable request.

## Abbreviations

DMFT: The decayed, Missing, and filled teeth
OR: Odds ratio
Cl: Confidence interval
*X*^2^: Chi-square test value
PPS: Probability Proportionate to Size Sampling
NDVI: Normalized Difference Vegetation Index
DEM: Digital Elevation Model
ANOVA: Analysis of variance
SSE: Sum of squares of error
SDH: Social determinants of health
