# Supplementary material for: Effect of dietary patterns on dental caries among 12–15 years-old adolescents: a cross-sectional survey
Source: BMC Oral Health. 2023 Nov 9;23:845. doi: 10.1186/s12903-023-03566-y (PMC10633925; doi:10.1186/s12903-023-03566-y)
Supplement: Supplementary file 1 — Additional file 1. Questionnaire for the first oral health epidemiological survey in Shanxi Province (Adolescents aged 12-15). [file 12903_2023_3566_MOESM1_ESM.pdf]

Questionnaire for the First Oral Health Epidemiological Survey in Shanxi Province (Adolescents aged 12-15)

The ID number of the respondent:□□□□□□□□□□□□

School: ____Grade: ____ Class: ____ **Name: ______ : Age:______**

Date of birth: ____________________ **Account type: ________**

Gender:□ Ethnic group:□ **Investigator No. :□**

Date: ______________

Dear students,

Hello everyone! In order to improve the oral health care of adolescents, we would like to know your thoughts and practices on oral health care. We have already obtained the permission of your guardian and teacher. This survey has nothing to do with your academic performance, and the results will not be told to parents or teachers. We hope you can answer the questions truthfully. thank you!

Requirements: please tick the "√" in front of the corresponding options in the choice questions.

Basic personal information:

1. Are you the only child in your family?（Choose only one answer）

   1)□Yes 2)□No

2. How many people live together in your family? **(Choose only one answer)**

   1)□2 2)□3 3)□4 4)□5 5)□6 or above

3. What is your father's highest education? **(Choose only one answer)**

   1)□Not going to school 2)□ Primary school 3)□Junior high school 4)□High school

   5)□Technical secondary school 6)□College 7)□Undergraduate 8)□Master's and above

   9)□Don't have a father or don't know

4. What is your mother's highest education? **(Choose only one answer)**

   1)□Not going to school 2)□ Primary school 3)□Junior high school 4) □High school

   5)□Technical secondary school 6)□College 7)□Undergraduate 8)□Master's and above

   9)□Don't have a mother or don't know

5. Where do you currently live and how many years have you lived there.

   ____

6. Do you have a history immigration?（Choose only one answer）

   1)□No 2)□Yes

Oral health knowledge:

1. Do you think the following statement is correct? **(Choose one answer for each subquestion)**

	Correct	Wrong	Don’t know
1) It is normal for your gums to bleed while brushing.	☐	☐	☐
2) Bacteria can cause gum inflammation.	☐	☐	☐
3) Brushing teeth will not help prevent gum inflammation.	☐	☐	☐
4) Bacteria can cause dental caries.	☐	☐	☐
5) Eating sugar can cause dental caries.	☐	☐	☐
6) Fluoride is not useful for protecting teeth.	☐	☐	☐
7) Pit and fissure closure can protect teeth .	☐	☐	☐
8) Oral diseases may affect your overall health.	☐	☐	☐
9) Electric toothbrushes are more effective than manual brushes	☐	☐	☐

Oral health attitude:

1. What do you think of the following statements? **(Choose one answer for each subquestion)**

	1<br>**Agree**	2<br>**Disagree**	3<br>**Doesn't matter**	4<br>**Don't know**
1) Oral health is important to my life	☐	☐	☐	☐
2) Regular oral check-ups are essential	☐	☐	☐	☐
3)Quality of teeth is determined at birth	☐	☐	☐	☐
4 Protecting teeth start with ourselves	☐	☐	☐	☐

2 .In the past 6 months, You suffer from oral problems while doing the following activities

	1 Strongly Agree	2 Agree	3 Neutral	4 Disagree	5 Strongly Disagree
1) Eating	☐	☐	☐	☐	☐
2) Speaking	☐	☐	☐	☐	☐
3) Brush teeth	☐	☐	☐	☐	☐
4) Doing the housework	☐	☐	☐	☐	☐
5) Studying	☐	☐	☐	☐	☐
6) Sleeping	☐	☐	☐	☐	☐
7) Smile with teeth	☐	☐	☐	☐	☐
8) Easily annoyed	☐	☐	☐	☐	☐
9) Social contact	☐	☐	☐	☐	☐

Oral health behaviors:

1. Do you brush your teeth? **(Choose only one answer)**

1)☐Brush my teeth. 2)☐Brush occasionally or never (do not answer Questions 2 to 1 1 if you choose this item)

2. How do you place your toothbrush after you brush your teeth? (Choose only one answer)

1)☐Brush head up 2)☐Brush head down 3)☐Placed at random
4)☐Closed place

3. How many times a day do you brush your teeth? **(Choose only one answer)**

1)☐2 times or more 2)☐Once a day 3)☐Not brushing every day

4.How long do you brush each time? (Choose only one answer)

1) ☐1 minute or less 2)☐1 - 2 minutes 3) ☐2-3 minutes 4)☐More than 3 minutes

5) ☐Non-fixed time 6)☐Don't know

5 Do you bleed when you brush your teeth? **(Choose only one answer)**

1)☐No. 2)☐ Once in a while 3) ☐ Every day 4) ☐Every time

6. Do you use toothpaste when you brush your teeth? **(Choose only one answer)**

1)☐Yes 2)☐No (do not answer Questions 7 to 8 if you choose this item)

3)☐Don't know (do not answer Questions 7 to 8 if you choose this item)

7. Do you use fluoride toothpaste when you brush your teeth? **(Choose only one answer)**

1)☐Yes 2)☐No 3)☐I don't know

8. Which of the following types of toothpaste do you use: **(Choose morethan one answer)**

1) ☐It doesn't matter. 2)☐Anti-acid desensitization

3)☐Chinese herbal medicine 4)☐Whitening

5)☐Antibacterial 6)☐Fluoride 7)☐I don't know

9. What is the main way you brush your teeth regularly? **(Choose only one answer)**

1)☐Vertical brush 2)☐A whipsaw horizontal brush

3)☐Spin and turn type 4)☐Horizontal flutter brush method

5)☐Not clear, not regular

10. How often do you change your toothbrush? **(Choose only one answer)**

1)☐Less than 3 months 2)☐3-6 months 3)☐6 months or more

4)☐Never replace your toothbrush as long as it's not broken

11. What are the bristles of your toothbrush? **(Choose only one answer)**

1)☐Hard 2)☐Moderate hardness and softness 3)☐Soft 4)☐Don't know

12. Do you use floss?(Choose only one answer)

1)☐No 2)☐Occasionally 3)☐ Every week 4)☐Every day

Other projects:

(1) Daily life

1. How many oral health courses did you take at school last semester?____(Please fill in a whole number, "N" if you do not know or refuse to answer)

2. Have you had toothache or discomfort in the past 12 months? **(Choose only one answer)**

1)☐Never 2)☐Sometimes 3)☐Often 4)☐Don't know

3. Have you been to the hospital for dental treatment? **(Choose only one answer)**

1)☐Yes 2)☐No (do not answer questions 4 to 10)

4. How long has it been since you last went to the hospital to see a dentist? **(Choose only one answer)**

1)☐Within 6 months (do not answer Questions 1 2 if you choose this item)

2)☐6 months to 12 months (do not answer Questions 1 2 if you choose this item)

3)☐Over 12 months (do not answer Questions 8 to 9 if you choose this item)

5. What was the main reason for your last visit to the hospital? **(Choose only one answer)**

1)☐Counseling and check 2)☐Prevention 3)☐Treatment 4) ☐ Don't know

6. Have you ever had an oral health check（choose only one answer）

1)☐Never (do not answer Questions 7 if you choose this item) 2:☐ For the school organization

3)☐ Once in a while 4）☐Regular inspection

7. How often do you get a dental checkup? **(Choose only one answer)**

1)☐Never 2)☐ < 6 months 3)☐ 6 and 12 months 4) ☐> 12 months 5)☐Not on a regular basis

8. How much did you spend on dental care in the hospital in the past year? _____**(Please fill in a whole number, "N" if you don't know or refuse to answer)**

9 Of the above dental costs,how much the percentage you will personally have to pay? _____%

(Please fill in a whole number, "N" for those who do not know or refuse to answer)

10. Where do you usually go to see a doctor for your oral disease? **(Choose more than one answer)**

1)☐Township street health centers 2)☐County/district hospitals 3)☐Specialized hospitals

4)☐Dental clinic

5)☐Municipal hospitals and above 6)☐Other 7)☐Don’t know

11. What i s the total income of your family members in the past 12 months? **(Choose only one answer)**

1)☐< $10,000 2)☐$10,000-$20,000 3)☐$20,000-$40,000 4)☐$40,000-$80,000.

5)☐$80,000-$160,000 6)☐> $160,000 7)☐Unclear/refused to answer

12. What is the reason you haven't seen a dentist in the past 12 months?(Multiple selections are available)

1)☐Nothing wrong with my teeth

2) ☐Not serious dental disease

3) ☐Don't have the time

4) ☐Having financial difficulties

5) ☐Don't reimbursed

6) ☐Can not find a dentist nearby

7) ☐Fear of infectious diseases

8) ☐Fear of dental pain

9) ☐It's hard to find a dentist that I can trust

10) ☐Difficulties in registering

11)Other reasons:________________________

(2)caries related factors

(common stems for questions 1-8) Approximately how often do you eat the following foods or drinks？

1. Desserts and confectionery (biscuits, cakes, bread, chocolate, sugary mouth candy, etc.)

1) ☐Never or hardly ever
2) ☐1~3 times a month
3) ☐Once a week
4) ☐2~6 times a week
5) ☐Once a day
6) ☐More than once a day

2. Sweet drinks (sugar water, carbonated drinks such as cola, fruit juices such as orange juice and apple juice, non-fresh fruit juices such as lemonade, etc.)

1) ☐Never or hardly ever
2) ☐1~3 times a month
3) ☐Once a week
4) ☐2~6 times a week
5) ☐Once a day
6) ☐More than once a day

3. Sugar-sweetened milk (yogurt, milk powder, tea, soy milk, coffee, etc.)

1) ☐Never or hardly ever

2) ☐1~3 times a month

3) ☐Once a week

4) ☐2~6 times a week

5) ☐Once a day

6) ☐More than once a day

4. Vegetables.

1) ☐Never or hardly ever

2) ☐1~3 times a month

3) ☐once a week

4) ☐2~6 times a week

5) ☐Once a day

6) ☐More than once a day

5. Fruits.

1) ☐Never or hardly ever

2) ☐1~3 times a month

3) ☐Once a week

4) ☐2~6 times a week

5) ☐Once a day

6) ☐More than once a day

6. Coarse grains(corn, purple rice, sorghum, oats, buckwheat, wheat bran)

1) ☐Never or hardly ever

2) ☐1~3 times a month

3) ☐Once a week

4) ☐2~6 times a week

5) ☐Once a day

6) ☐More than once a day

7. Protein foods(beans, eggs, meat, fish, animal offal, etc.)

1) ☐Never or hardly ever

2) ☐1~3 times a month

3) ☐Once a week

4) ☐2~6 times a week

5) ☐Once a day

6) ☐More than once a day

8. Are there any supermarkets and food stores in your school? (**Choose only one answer**)

   1)☐No 2)☐Yes

(3) Related factors of oral mucosal disease

1. Your smoking status: **(choose one answer for each sub question)**

1)☐Never smoked 2)☐smoking 3) ☐Used to smoke, now quit

Smokers now	1)☐ 10 / day or less 2)☐> 10 sticks/day,≤1 pack/day<br>3)☐> 1 pack/day
People who have given up smoking	1)☐Quitting smoking 1 month or less 2)☐Quit smoking > 1 month, ≤6 months<br>3)☐Quit smoking > 6 months,≤1 year 4)☐Quit smoking > 1 year

(4) Related factors of malocclusion

1. Do you think your teeth are straight? **(Choose only one answer)**

1)☐Yes 2)☐No 3)☐I don't know

2 Do you suffer from nasopharyngeal disease? **(Choose more than one answer)**

1)☐No. 2)☐Tonsillitis 3)☐Rhinitis 4)☐Pharyngitis

5)☐Adenoid hypertrophy 6)☐Don't know

3 Do you have a history of oral and nasal surgery? **(Choose only one answer)**

1)☐No 2)☐Yes 3)☐Don't know

4. Do you have any of the following bad oral habits? **(Choose more than one answer)**

1) ☐No 2)☐Lip biting habit 3)☐Bite hard objects such as pencils 4)☐Finger sucking habit

5) ☐sticking out the tongue habit 6)☐Chin protruding habit 7)☐holding gills 8) ☐Breathe with open your mouth

9) ☐Unilateral mastication 10)☐snore 11)☐Clenched teeth habit 12)☐The habit of opening your mouth wide

13)☐Lick your lips 14) ☐Don't know

5. According to family or friends, do you grind your teeth at night? **(Choose only one answer)**

1)☐No 2)☐Yes 3）☐I don’t know

6. Does your family have irregular teeth?Relationship with you? **(Choose only one answer)**

1)☐None (do not answer Questions 7) 2)☐Dad 3)☐Mother

4)☐Grandpa. 5)☐Grandma

6) ☐Unclear (do not answer Questions 7 if you choose this item)

7. If there are irregular teeth or any of the following in the family, the specific type is: **(Multiple answers are optional)**

1)☐No. 2)☐Mandibular protrusion 3)☐Maxillary protrusion

4)☐Mouth protruding 5)☐Crowded teeth 6)☐Cleft lip and palate

7)☐Don't know 8)☐Teeth sparse

8. Do you think you need orthodontic treatment? **(Choose one only answer)**

1)☐Yes 2)☐No 3)☐Don't know

9. Have you ever worn or are currently wearing an appliance? **(Choose only one answer)**

1)☐Never 2)☐Used to wear it

3)☐Are wearing

(5) Factors related to dental trauma

1. Have you ever bruised or fallen your teeth? **(Choose only one answer)**

1)☐Have wounded 2)☐No injury (do not answer Questions 2 if you choose this item)

3)☐Can't remember (do not answer Questions 2 if you choose this item)

2. Where did you hurt your tooth? **(Choose more than one answer)**

1) ☐On campus 2) ☐3) At home ☐Other place:_______

(6) Related factors of temporomandibular joint diseases

1. Do you have the following symptoms of temporomandibular joint disease? **(Choose only one answer)**

1)☐No 2)☐ an abnormal noise 3)☐Pain 4)☐Rigidity

(7) Self-evaluation status:

1. How do you rate your general health? **(Choose only one answer)**

1)☐ Very healthy 2)healthy 3)☐ Sub-healthy 4)☐Unhealthy

2. How do you rate the condition of your teeth and mouth? **(Choose only one answer)**

1)☐Very healthy 2)☐healthy 3)☐ Sub-healthy 4)☐Unhealth

Thank you very much!

Please follow the instructions of the staff to complete the oral health examination.At the same time, we respect your choice to opt out of this oral epidemiological survey at any time.If you have any suggestions about our investigation, please point them out.
